# Long Non-Coding RNAs as Regulators of Interactions between Cancer-Associated Fibroblasts and Cancer Cells in the Tumor Microenvironment

**DOI:** 10.3390/ijms21207484

**Published:** 2020-10-11

**Authors:** Young-Ho Ahn, Jeong Seon Kim

**Affiliations:** 1 Department of Molecular Medicine, College of Medicine, Ewha Womans University, Seoul 07804, Korea; jeongseonkim821@gmail.com; 2 Inflammation-Cancer Microenvironment Research Center, College of Medicine, Ewha Womans University, Seoul 07804, Korea

**Keywords:** long non-coding RNAs, cancer-associated fibroblasts, tumor microenvironment

## Abstract

Long non-coding RNAs (lncRNAs) regulate diverse physiological and pathological processes via post-transcriptional, post-translational, and epigenetic mechanisms. They are also involved in tumor initiation, progression, and metastasis by functioning as key players in the tumor microenvironment. Cancer-associated fibroblasts (CAFs) promote tumor initiation, progression, metastasis, drug resistance, and immunosuppression, which can be modulated by lncRNAs. LncRNAs regulate the intrinsic properties of CAFs or cancer cells intracellularly or function extracellularly through exosomal secretion. In-depth studies on the mechanisms of lncRNA functions will enable their clinical use as diagnosis/prognosis markers and therapeutic targets in cancer treatment.

## 1. Introduction

With the development of next-generation sequencing technology, tens of thousands of long non-coding RNAs (lncRNAs) have been discovered. According to LNCipedia, as of September 2020, 127,802 transcripts and 56,946 lncRNA genes have been annotated in the human genome (https://hg38.lncipedia.org/). LncRNAs are transcripts longer than 200 nucleotides in length lacking protein-coding ability that function as crucial physiological and pathological regulators via post-transcriptional, post-translational, and epigenetic mechanisms. Growing bodies of evidence indicate that numerous lncRNAs are also involved in tumor initiation, progression, and metastasis. In particular, the role of lncRNAs in the tumor microenvironment is rapidly emerging. This review focuses on the regulation, function, mechanisms of action, and clinical implications of lncRNAs in cancer-associated fibroblasts (CAFs), the major components of the tumor microenvironment.

## 2. Functions of lncRNAs

LncRNAs play crucial roles in many biological and cellular processes and are related to diseases including cancer. They function through the direct or indirect interaction with genomic DNA, mRNA, microRNAs (miRNAs), and proteins; however, the exact molecular mechanisms underlying lncRNA functions are largely unknown. Unlike protein-coding genes or miRNAs, lncRNAs have no particular sequence or conserved secondary structures to deduce their functions. However, even though lncRNAs do not share a common working mechanism, lncRNA functions can be categorized as signals, decoys, scaffolds, or guides depending on the relationship with the interacting partner.

### 2.1. Signal lncRNAs

Signal lncRNAs, serving as “molecular signals”, play important roles in signal regulation and response to various stimuli. These types of lncRNAs are expressed in a spatial- and temporal-specific pattern. KCNQ1 overlapping transcript 1 (*KCNQ1OT1*) is known to act as a signal lncRNA by interacting with histone methyltransferase G9a and polycomb repressive complex 2 (PRC2). Through the recruitment of chromatin remodeling complex, *KCNQ1OT1* controls lineage-specific transcriptional silencing during embryonic liver development [1]. The p21-associated ncRNA DNA damage-activated (*PANDA*) lncRNA is induced in a p53-dependent manner in osteosarcoma cells. When activated by DNA damage, p53 binds to the *CDKN1A* locus and transactivates *PANDA*. *PANDA* then interacts with the transcription factor NF-YA to inhibit the expression of proapoptotic genes [2].

### 2.2. Decoy lncRNAs

Decoy lncRNAs restrict the function of regulatory factors by presenting decoy-binding sites. These lncRNAs modulate transcription by secluding regulatory factors such as transcription factors, enzymes, and miRNAs. Telomeric repeat-containing RNA (*TERRA*) has been shown to form an integral part of telomeric heterochromatin. *TERRA* interacts with both telomerase RNA hTR and telomerase reverse transcriptase protein TERT and acts as a competitive inhibitor of telomerase [3]. Growth arrest-specific 5 (*GAS5*), a lncRNA with hairpin structures, accumulates during nutrient- or growth factor-deprivation. *GAS5* competes with glucocorticoid-response elements in binding to glucocorticoid receptors, thereby suppressing glucocorticoid-mediated transcription [4]. In addition, PTEN pseudogene 1 (*PTENP1*) has been reported to function as a competing endogenous RNA (ceRNA) for miRNAs. *PTENP1* upregulates PTEN expression by sponging PTEN-targeting miRNAs (e.g., miR-17, miR-21, miR-214, miR-19, and miR-26) [5], leading to tumor suppression [6].

### 2.3. Scaffolds lncRNAs

LncRNAs can act as molecular scaffolds bringing two or more proteins together to form nucleoprotein complexes and be involved in gene activation, gene repression, and chromatin modification [7]. For example, HOX transcript antisense intergenic RNA (*HOTAIR*) serves as a scaffold for two distinct histone modification complexes. It was shown that the 5′-domain of *HOTAIR* binds to PRC2 to promote gene repression, and the 3′-domain binds to the LSD1/coREST/REST complex, which demethylates lysine 4 of histone H3 (H3K4) to repress gene activation [8]. In glioblastoma, EGFR-induced lncRNA *NEAT1* interacts with EZH2 on the promoter regions of negative regulators (*CTNNBIP1*, *GSK3B*, and *AXIN2*) in the Wnt/β-catenin pathway, which leads to tumor progression [9]. Moreover, antisense non-coding RNA at the INK4 locus (*ANRIL*) is upregulated in adult T-cell leukemia and associates with EZH2 and the RelA/p65 subunit of NFκB, which enhances NFκB signaling and promotes cancer cell proliferation. An association between *ANRIL* and EZH2 also increases histone methylation on the promoter region of p21, which causes transcriptional silencing of p21, a cell cycle inhibitor [10].

### 2.4. Guide lncRNAs

**Figure 1:**
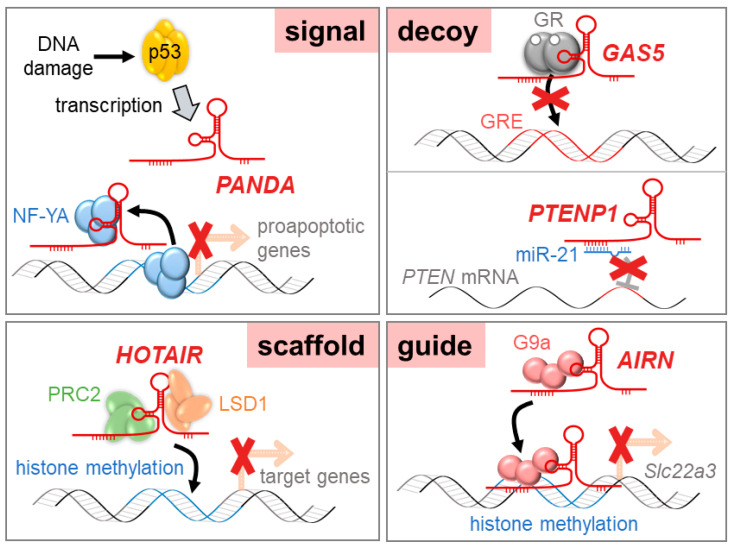
Diverse functional mechanisms of long non-coding RNAs (lncRNAs).

Certain types of lncRNAs interact with target molecules and guide them to the proper chromosomal localization. Guide lncRNAs regulate the transcription of target genes at their genomic loci. During the mouse embryonic development of the heart and body wall, fetal-lethal non-coding developmental regulatory RNA (*Fendrr*) guides PRC2 to promoter regions of target genes (transcription factors for mesoderm differentiation) to increase H3K4 trimethylation, leading to attenuation of target gene expression [11]. In mouse placenta, antisense of the IGF2R non-protein coding RNA (*AIRN*) has been shown to silence the transcription of cis-linked genes via chromatin interaction at their promoter regions. Accumulated *AIRN* on the *Slc22a3* promoter recruits G9a, which induces targeted histone methylation and allelic silencing [12]. LncRNA functions are shown schematically in Figure 1.

## 3. Cancer-Associated Fibroblasts (CAFs)

The tumor microenvironment provides a tumor with a supportive niche to promote tumor progression and metastasis. Among the components of the tumor microenvironment are CAFs, lymphocytes, macrophages, and vascular endothelial cells, as well as the extracellular matrix. Stromal CAFs are major players in the tumor microenvironment that facilitate tumor progression and metastasis via direct or indirect interactions with neighboring cancer cells.

### 3.1. Origins of CAFs

Quiescent fibroblasts can differentiate into CAFs upon stimulation by diverse external cues such as cytokines/chemokines, growth factors, reactive oxygen species, hypoxia, and non-coding RNAs. TGF-β, a strong inducer of desmoplastic reaction and fibrosis, activates fibroblasts in pancreatic adenocarcinoma [13]. TGF-β stimulates the expression of myofibroblast markers and metabolic reprogramming in stromal fibroblasts of breast cancer [14]. Pro-inflammatory signals from immune cells and tumor cells educate normal fibroblasts into becoming CAFs via IL-1β and the NFκB pathway [15]. In prostate cancer, TGF-β derived from cancer cells activates fibroblasts via NADPH oxidase 4-mediated reactive oxygen species signaling [16]. Hypoxia causes the epigenetic reprogramming of breast fibroblasts, resulting in them gaining CAF-like pro-glycolytic phenotypes [17]. In addition, numerous miRNAs have been reported to be involved in the differentiation of normal fibroblasts into CAFs [18].

Besides simply differentiating from normal fibroblasts, CAFs are able to originate from different types of cells residing in the tumor microenvironment (e.g., epithelial cells, endothelial cells, pericytes, and adipocytes) or migrating from distant sites (e.g., mesenchymal stem cells) [19,20,21]. Through the epithelial-to-mesenchymal transition (EMT) process, epithelial cells adjacent to cancer cells can differentiate into CAFs [22,23]. TGF-β-induced endothelial-to-mesenchymal transition in endothelial cells also contributes to the accumulation of CAFs in the tumor microenvironment [24]. Vascular pericytes stimulated by PDGF-BB acquire stromal fibroblast features [25]. Even adipose tissue-derived stem cells around breast cancer cells differentiate toward CAFs in response to TGF-β and Smad3 signaling [26].

Cells recruited from outside the tumor microenvironment can be converted to CAFs. In gastric cancer, it was shown that quite a significant population of CAFs originated from bone marrow-derived mesenchymal stem cells [27]. SDF-1α/CXCR4 and TGF-β signaling promote the differentiation of bone marrow-derived mesenchymal stem cells into myofibroblasts, which are then recruited to the tumor microenvironment. In prostate cancer, mesenchymal stem cells are recruited from bone marrow contact with prostate cancer cells in the tumor microenvironment and then trans-differentiated into CAF-like cells via TGF-β signaling [28]. CAFs can also derive from hematopoietic stem cells [29], which create a pro-tumorigenic microenvironment [30].

### 3.2. Effects of CAFs on Cancer Development

CAFs are involved in the entire cancer developmental process, from tumorigenesis to progression and metastasis. Most CAFs exert pro-tumorigenic effects, while certain subpopulations of CAFs have anti-tumorigenic effects [19].

CAFs promote tumor initiation and proliferation by secreting growth factors and cytokines. CAFs isolated from ovarian cancer patients promote cancer cell proliferation through the paracrine secretion of FGF-1, which is mediated by FGFR4 [31]. CAF-derived HGF also activates the proliferation of ovarian cancer cells thorough c-Met/PI3K/Akt signaling [32]. In endometrial cancer, CAFs promote cancer cell proliferation via the secretion of CXCL12 or IL-6 [33,34]. CAF-derived CCL2 stimulates the sphere-forming activity and self-renewal of breast cancer stem cells through NOTCH signaling [35]. In non-small cell lung cancer, CAFs promote the growth and stemness of lung cancer stem cells via IGF-II secretion and Nanog expression [36].

CAFs in the tumor microenvironment promote the progression of tumors in various ways. CAFs secrete IL-6 and activate STAT3 to induce the EMT, migration, and invasion of bladder cancer cells [37]. CAF-derived IL-6 also induces EMT, migration, and clonogenicity in esophageal adenocarcinoma [38]. CAFs promote the EMT of recipient lung cancer cells through the exosomal delivery of SNAIL, an EMT-inducing transcription factor [39]. The transfer of miR-181d-5p from CAFs to breast cancer cells via exosomes then induces EMT by the direct targeting of CDX2, a transcription factor for HOXA5 [40]. TGF-β from CAFs promotes EMT and invasion in bladder cancer cells through the regulation of lncRNA *ZEB2NAT* [41], which will be described in more detail below. Consequently, CAFs can induce cancer cell invasion through direct physical interaction [42].

Galectin-1 originating from CAFs in gastric cancer promotes proliferation, migration, and tube formation of vascular endothelial cells and enhanced tumor angiogenesis in vivo [43]. The upregulation of WNT2 in colorectal CAFs stimulates the migration and invasion of endothelial cells and promotes angiogenesis through the production of pro-angiogenic factors [44]. VEGF-bound small extracellular vesicles are secreted by CAFs to enhance tumor angiogenesis [45]. In addition, CAFs induce biomechanical deformation of the extracellular matrix and promote tumor-supportive vascularization [46].

CAFs also modulate glycolytic metabolism in cancer cells. Downregulation of focal adhesion kinase in CAFs increases CCL6 and CCL12 expression, leading to elevated glycolysis in cancer cells [47]. CAFs educated by neighboring cancer cells reversely secrete cytokines to stimulate glycogen metabolism in cancer cells, which accelerates energy production and tumor progression [48]. Epigenetic changes in prostate CAFs drive the synthesis of glutamine, which is then transported to adjacent cancer cells to promote energy production, differentiation, and the aggressiveness of prostate cancer cells [49]. Interestingly, CAFs have been reported to transfer their own mitochondrial DNA via exosomes or mitochondria via intercellular bridges to adjacent cancer cells, resulting in metabolic reprogramming and enhanced malignancy [50,51].

The tumor microenvironment provides cancer cells with shelter from the attack of anticancer drugs [52]. The interplay between CAFs and cancer cells contributes significantly to drug resistance. CAFs reduce cisplatin-induced apoptosis in bladder cancer cells by activating signaling cascades, leading to Bcl-2 upregulation [53]. CAFs secrete IL-6, which activates the JAK/STAT3 pathway and reduces the susceptibility of gastric cancer cells to 5-fluorouracil [54]. In lung and ovarian cancers, CAF-derived HGF activates PI3K/Akt signaling and suppresses the paclitaxel-induced apoptosis of cancer cells [32,55]. Neuregulin 1 from CAFs induces HER3 activation in prostate cancer cells, which is responsible for reduced sensitivity to androgen deprivation therapy [56]. Moreover, CAFs release extracellular vesicles containing Annexin A6, which induces FAK-YAP activation and drug resistance in gastric cancer cells [57].

In addition, CAFs are involved in immunosuppression and tumor-promoting inflammation through interactions with other stromal cells. In breast cancer, the glycoprotein Chitinase-3-like-1 secreted from CAFs enhances the recruitment and M2-type polarization of macrophages, leading to immunosuppression and the promotion of tumor growth [58]. In lung squamous cell carcinoma, CAFs recruit monocytes to the tumor microenvironment by secreting CCL2 and facilitate their differentiation into immunosuppressive myeloid-derived suppressor cells [59]. Furthermore, CAF-driven antigen presentation leads to the depletion of CD8^+^ T cells via PD-L2 and FasL within the tumor microenvironment [60].

## 4. LncRNAs that Regulate the Interaction between CAFs and Cancer Cells

As described above, CAFs and cancer cells interact together directly or indirectly, and they influence each other to promote tumor progression and metastasis. Reviewing the recently accumulated literature reveals that lncRNAs are also important mediators between cancer cells and CAFs. The lncRNAs function within cells (CAFs and cancer cells) or are transported to other cells via exosomes (from CAFs to cancer cells and vice versa).

### 4.1. LncRNAs Working within CAFs

The lncRNA profiling of CAFs has been attempted in various cancers to examine overall expression changes and to select novel candidates working in the tumor microenvironment. High-throughput methods to profile lncRNA expression levels include quantitative reverse transcription (qRT)-PCR-based arrays, microarrays, and RNA sequencing, and each of these methods has advantages and disadvantages [61]. Compared with RNA sequencing, PCR arrays and microarrays are limited to known transcripts. However, microarrays generally do not involve PCR-based amplifications, which can cause unwanted bias.

Teng et al. performed lncRNA profiling to identify differentially expressed lncRNAs between CAFs and adjacent normal fibroblasts obtained from patients with non-small cell lung cancer [62]. They performed lncRNA microarrays using three matched pairs of CAFs and normal fibroblasts. A total of 766 lncRNAs showed differential expression patterns in CAFs: 322 lncRNAs were upregulated and 444 lncRNAs were downregulated. Gene ontology (GO) and Kyoto Encyclopedia of Genes and Genomes (KEGG) pathway analyses revealed that upregulated lncRNAs in CAFs are involved in immune network pathways such as type I interferon signaling, defense response to viruses, NOD-like receptor signaling, Wnt signaling, and toll-like receptor signaling. These results suggest that CAF-specific lncRNAs mediate an immune response during lung cancer progression.

In ovarian cancer, Colvin et al. profiled CAF-specific lncRNAs associated with patient overall survival [63]. Using microarrays and Kaplan–Meier survival analysis, they identified ten CAF-specific lncRNAs: nine lncRNAs (*CRNDE*, *DANCR*, *LOC642852*, *MALAT1*, *MEG3*, *MGC2752*, *NEAT1*, *TP73-AS1*, and *XIST*) were associated with shorter survival, and one (*MIR155HG*) was associated with longer survival. A co-expressed interactome with protein-coding genes and pathway (GO and KEGG) analyses showed that *MIR155HG*-related genes are associated with the immune system, with involvement in processes such as T-cell activation, antigen presentation, and leukocyte migration. Gene clusters involving *DANCR*, *LOC642852*, *MALAT1*, *MEG3*, *MGC2752*, *TP73-AS1*, and *XIST* are associated with metabolic processes and autophagy. The study showed that CAF-specific lncRNAs are linked to the survival of patients with ovarian cancer, and these lncRNAs regulate the tumor microenvironment to boost tumor growth and immune evasion.

Beyond global lncRNA profilings, in-depth studies on the roles and working mechanisms of individual lncRNAs in cancer development have also been conducted. In oral squamous cell carcinoma, RNA sequencing was performed to profile the changes of stromal lncRNAs during CAF transformation from normal fibroblasts [64]. Among those lncRNAs upregulated in CAFs, an intergenic lncRNA *FLJ22447* (referred to as *Lnc-CAF* in the paper) proved to be required to maintain the stromal features of CAFs. The working mechanism of *FLJ22447* was investigated through siRNA-mediated knockdown or overexpression. *FLJ22447* increased the stability of IL-33 by preventing autophagic degradation, which promotes CAF reprogramming and tumor growth. In terms of clinical relevance, high *FLJ22447* expression was associated with a high TNM stage and poor patient survival. Based on these findings, *FLJ22447* promotes CAF activation and tumor progression in oral squamous cell carcinoma.

CAF-specific lncRNAs can be molecular targets of anticancer drugs. Sipi soup, a traditional Chinese medicine against infection and inflammation, prevents cancer cell-guided CAF activation and cancer progression in cervical cancer [65]. Through lncRNA microarray profiling, HIPK1 antisense RNA (*HIPK1-AS*) was shown to be induced in CAFs after treatment with conditioned medium from HeLa cells, and this was attenuated by Sipi soup. The shRNA-mediated knockdown of *HIPK1-AS* blocked the activation of CAFs by HeLa conditioned medium. In addition, *HIPK1-AS* was expressed at higher levels in cervicitis and cervical squamous cell carcinoma than in normal cervical cells. Upregulated *HIPK1-AS* in tumor stroma may cause inflammation and progression of cervical cancer; therefore, *HIPK1-AS* could be a good therapeutic target.

Another study in oral squamous cell carcinoma showed a more detailed working mechanism of a lncRNA in CAFs [66]. LncRNAs with significantly differential expression in CAFs from oral squamous cell carcinoma tissues compared with fibroblasts from normal tissues were identified using RNA sequencing: 29 lncRNAs were upregulated and 17 were downregulated. Among those, *TIRY* was highly expressed in CAFs. Bioinformatic and consequent in vitro approaches (RNA pull-down assays) found that *TIRY* can function as an endogenous sponge against miR-14. In oral squamous cell carcinoma tissues, *TIRY* expression was inversely correlated with miR-14 expression. Patients with high *TIRY* expression had poorer overall and progression-free survival than those with low *TIRY* expression. CAFs with higher *TIRY* expression present strong EMT features and promote the invasion and metastasis of co-cultured cancer cells. Interestingly, *TIRY* in CAFs suppressed exosomal delivery of miR-14 to neighboring cancer cells, which caused the upregulation of WNT3A, a miR-14 target, and activation of Wnt/β-catenin signaling in cancer cells. *TIRY* induces EMT in CAFs and promotes the progression and metastasis of oral squamous cell carcinoma by blocking miR-14 delivery; therefore, *TIRY* could be a prognostic and therapeutic target.

### 4.2. LncRNAs Working within Cancer Cells

A vast amount of research has been reported on the roles and mechanisms of lncRNAs in various types of cancer cells [67]. In this section, we focus on the studies of lncRNAs within cancer cells interacting with CAFs in the tumor microenvironment.

In urinary bladder cancer, conditioned medium from CAFs induced the EMT, migration, and invasion of cancer cells [41]. The main player within the conditioned medium was TGF-β, a strong EMT inducer [68]. To discover lncRNA mediators, a PCR array with 72 probes detecting cancer-related lncRNAs was performed in bladder cancer cells treated with conditioned medium from CAFs: three upregulated and six downregulated lncRNAs were identified. *ZEB2NAT*, a natural antisense transcript to the ZEB2 gene (also known as *ZEB2-AS1*), was among the upregulated lncRNAs. TGF-β induced *ZEB2NAT* transcription, and *ZEB2NAT* induced the EMT, migration, and invasion of bladder cancer cells. Inversely, *ZEB2NAT* depletion by siRNAs restored the enhanced invasion by conditioned medium from CAFs or TGF-β. *ZEB2NAT* is known to positively regulate ZEB2 expression via a post-transcriptional mechanism [69]. In bladder cancer samples, TGF-β, *ZEB2NAT*, and ZEB2 expression levels correlate positively and increase significantly in invasive tumors compared with non-invasive tumors. Therefore, *ZEB2NAT* responds to EMT and invasion signals from CAFs and mediates EMT and invasion in bladder cancer cells.

CXCL14 is known to mediate CAF-induced pro-tumorigenic effects (migration, invasion, and metastasis) in ovarian cancer [70]. CXCL14 is highly expressed in tumor stroma compared with normal stroma and is negatively correlated with the overall survival of patients with ovarian cancer. Using lncRNA microarrays, it was found that *LINC00092* was one of the lncRNAs upregulated in ovarian cancer cells after CXCL14 treatment. *LINC00092* knockdown by siRNAs reduced invasion and induced anoikis in ovarian cancer cells, and suppressed metastasis and extended mouse survival duration in a xenograft model. *LINC00092* knockdown also causes a reduction in glycolysis metabolites, such as lactate. RNA pull-down assays proved that *LINC00092* binds directly to a glycolytic enzyme, PFKFB2. In ovarian cancer patients, a positive correlation between *LINC00092* and PFKFB2 protein expression was observed, which suggests that *LINC00092* directly interacts with PFKFB2, maintains PFKFB2 enzyme stability, and modulates glycolysis during ovarian cancer progression and metastasis. Reciprocally, the glycolytic phenotype augmented by *LINC00092* and PFKFB2 in ovarian cancer cells helps CAFs to remain activated and facilitates metastasis in the tumor microenvironment.

Another lncRNA, urothelial carcinoma-associated 1 (*UCA1*), was also reported to be induced in glioma cells by paracrine CXCL14 secretion from glioblastoma-associated stromal cells [71]. *UCA1* positively regulates PFKFB2 expression in glioma cells by functioning as a sponge against miR-182, which directly targets PFKFB2. Modulating the interaction between glioma cells and glioblastoma-associated stromal cells, the CXCL14-*UCA1*-miR-182-PFKFB2 axis promotes glycolysis and the invasion of glioma. In colorectal cancer cells, *UCA1* expression is induced by conditioned medium from CAFs [72]. *UCA1* is involved in the promotion of the proliferation, migration, invasion, and EMT of colorectal cancer cells, which is mediated by blocking miR-143 and relieving KRAS inhibition [73].

Functioning as a ceRNA or a miRNA sponge is one of the major mechanisms of lncRNAs, as mentioned above. Cancer susceptibility candidate 9 (*CASC9*) is another example of a ceRNA in cervical cancer [74]. TGF-β released from CAFs elevates *CASC9* expression in cervical cancer cells. *CASC9* is increased in cancer tissues compared to normal tissues, and cervical cancer patients with low *CASC9* expression have better overall survival rates than those with high *CASC9* expression. *CASC9* expression levels were also associated with cancer pathological stages. Through RNA immunoprecipitation (IP), miRNA pull-down, and luciferase assays, it was found that *CASC9* competitively binds to miR-215 and releases its target *TWIST2* from suppression. Thus, signaling from CAFs (TGF-β) contributes to cervical cancer progression via the regulation of *CASC9*/miR-215/*TWIST2* signaling in cancer cells.

In breast cancer, TGF-β has been reported to be a key cytokine mediating the crosstalk between CAFs and cancer cells [75]. TGF-β is enriched in conditioned medium from CAFs and is required for CAFs to induce EMT in breast cancer cells. TGF-β stimulation encourages cancer cells to express more *HOTAIR*, which is a well-known epigenetic silencer that promotes tumor progression and metastasis [76]. Downstream of TGF-β, Smad2/3/4 transactivates *HOTAIR* by directly binding to its promoter region. *HOTAIR*, in turn, activates the EMT, migration, and invasion of breast cancer cells by the epigenetic suppression of *EGR1* and *CDK5RAP1* promoters. In addition, *HOTAIR* expression is higher in metastatic breast carcinoma than in ductal carcinoma in situ. Collectively, the TGF-β/*HOTAIR* axis controls breast cancer development and metastasis by facilitating communication between CAFs and breast cancer cells in the tumor microenvironment.

CAFs in the tumor microenvironment endow cancer cells with therapy resistance [77]. In oral squamous cell carcinoma, midkine, a member of the heparin-binding growth factor family, was shown to mediate CAF-induced cisplatin resistance in cancer cells [78]. Midkine released from CAFs can block cisplatin-induced cancer cell death. CAF-derived midkine enhances *ANRIL* expression in cancer cells, which promotes cell proliferation and cisplatin resistance. *ANRIL* upregulates the expression of ATP-binding cassette transporter proteins and thus increases the efflux of cancer drugs. Moreover, high *ANRIL* expression is associated with high TNM stage and lymph node metastasis in patients with oral squamous cell carcinoma.

In esophageal squamous cell carcinoma, CAFs confer cancer cells with resistance to radiotherapy [79]. The expression of DNM3 opposite strand/antisense RNA (*DNM3OS*), one of the highly expressed lncRNAs screened by PCR array, increases in radioresistant esophageal cancer cells compared with parental cells. *DNM3OS* expression was also higher in tumor tissues than in normal tissues and was associated with tumor stage. Conditioned medium derived from CAFs stimulated *DNM3OS* expression in cancer cells via PDGFβ/PDGFRβ signaling, which modulates the irradiation-induced DNA damage response. Transcriptionally, FOXO1, a downstream transcription factor of PDGFβ/PDGFRβ signaling, binds to the promoter region of *DNM3OS* and upregulates its expression. CAF-induced lncRNA *DNM3OS* can be a therapeutic target to restore radio-resistance in esophageal squamous cell carcinoma.

### 4.3. Exosomal lncRNAs

Exosomes or extracellular vesicles function as essential mediators of cell-to-cell communication in the tumor microenvironment [80,81]. They transport specific cargo molecules including proteins, mRNAs, miRNAs, and lncRNAs. Recent findings have demonstrated that various exosomal lncRNAs are involved in the interaction between CAFs and cancer cells and contribute to cancer progression, drug resistance, cancer stemness, and metastasis.

In a mouse model of colorectal cancer, RNA sequencing was performed to identify differentially expressed transcripts in tumor tissues compared with normal colon tissues [82]. LncRNA *H19* was highly expressed in tumor tissues and increased according to cancer development stages and metastasis status. *H19* overexpression in colorectal cancer cells promotes cancer stem cell-like phenotypes and oxaliplatin resistance. RNA fluorescence in situ hybridization revealed that *H19* was predominantly expressed in tumor stroma, and CAFs expressed higher levels of *H19* than normal fibroblasts. Interestingly, *H19* is enriched in CAF-derived exosomes and is transported to cancer cells. In colon cancer cells, transported *H19* functions as a ceRNA for miR-141, which targets β-catenin. Through the exosomal transport of *H19*, CAFs activate the Wnt/β-catenin signaling in colorectal cancer cells, facilitating cancer stemness and drug resistance.

Colorectal cancer-associated lncRNA (*CCAL*), which was found to be upregulated in colorectal cancer through PCR arrays [83], was also shown to be involved in drug resistance. *CCAL* is highly expressed in oxaliplatin-resistant colorectal cancer cells and promotes drug resistance [84]. Just like *H19*, *CCAL* is highly enriched in CAF-derived exosomes, and exosomal *CCAL* has been observed to be transferred from CAFs to cancer cells, promoting drug resistance. *CCAL* in cancer cells activates Wnt/β-catenin signaling by modulating β-catenin mRNA stability. Through RNA IP and RNA pull-down assays, *CCAL* was shown to bind directly to HuR, an RNA-binding protein that increases β-catenin mRNA stability. Thus, exosomal *CCAL* secreted from CAFs promotes the drug resistance of colorectal cancer cells by the regulation of HuR and β-catenin.

In vulvar squamous cell carcinoma tissues, *UCA1* was highly expressed compared with normal tissues [85]. *UCA1* expression is associated with tumor stages and lymph node metastasis in vulvar squamous cell carcinoma. Again, high *UCA1* expression was detected in exosomes secreted from CAFs, and exosomal *UCA1* was transported to cancer cells. *UCA1* endowed cancer cells with resistance to cisplatin by functioning as a miRNA sponge against miR-103a which targets *WEE1*, a cell-cycle kinase regulating G2-M checkpoint. This study shows that CAFs promote the cisplatin resistance of cancer cells via exosomal *UCA1* transport.

Exosomes released from CAFs derived from breast cancer patients were found to promote the growth and change the metabolic pathways of cancer cells. This was mediated by small nucleolar RNA host gene 3 (*SNHG3*) enclosed in the exosomes [86]. *SNHG3* serves as a miRNA sponge against miR-330-5p and releases *PKM* from direct targeting by miR-330-5p. PKM is a glycolytic enzyme that converts phosphoenolpyruvate to pyruvate and is involved in cancer cell proliferation and metabolism [87]. Therefore, the exosomal transport of *SNHG3* from CAFs to neighboring cancer cells modulates the miR-330-5p/*PKM* axis and then stimulates cell proliferation and metabolism reprogramming in breast cancer cells.

Conversely, cancer cells secrete exosomes containing lncRNAs to induce reprogramming and differentiation of CAFs in the tumor microenvironment. Exosomes secreted from mouse melanoma cells were found to stimulate the expression of CAF-specific markers (α-SMA and FAP) and the migratory activity of fibroblasts (NIH/3T3) [88]. In melanoma-derived exosomes, the lncRNA *Gm26809* was significantly enriched. *Gm26809* is a key player in mediating the effect of melanoma-derived exosomes on the reprogramming of normal fibroblasts into CAFs. Reprogrammed fibroblasts, in turn, promote melanoma cell proliferation and migration. Supplemented with further investigation on the exact working mechanisms, exosomal *Gm26809* could be a good target for melanoma treatment.

**Figure 2:**
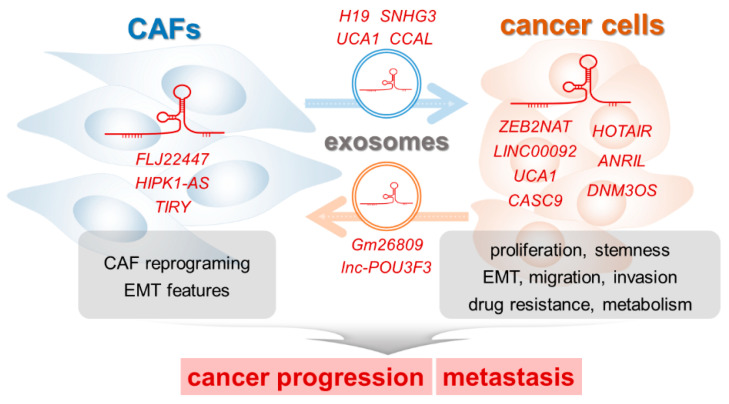
LncRNAs influence the interaction between CAFs and cancer cells in the tumor microenvironment.

Esophageal squamous cell carcinoma cancer cell-secreted exosomes upregulate the expression of CAF-specific markers in normal fibroblasts when compared with normal cell-secreted exosomes [89]. Among 14 esophageal squamous cell carcinoma-related lncRNAs, *lncRNA POU3F3* (*lnc-POU3F3*) in exosomes facilitates the reprogramming of normal fibroblasts to CAFs. The *lnc-POU3F3* lncRNA is transferred from cancer cells to fibroblasts via exosomes and activated fibroblasts. Fibroblasts activated by exosomal *lnc-POU3F3*, in turn, promote the proliferation and cisplatin resistance of cancer cells through the paracrine secretion of an inflammatory cytokine, IL-6. In esophageal squamous cell carcinoma patients, high plasma *lnc-POU3F3* levels are associated with resistance to chemoradiotherapy, suggesting that plasma exosomal *lnc-POU3F3* expression can be used as a liquid biopsy marker predicting the prognosis of patients with esophageal squamous cell carcinoma cancer. LncRNAs regulating the interaction between CAFs and cancer cells are summarized in Table 1 and Figure 2.

## 5. Conclusions and Perspectives

In the tumor microenvironment, cancer cells interact with various types of cells, such as lymphocytes, macrophages, endothelial cells, and CAFs. CAFs occupy an important position in the tumor microenvironment: they modulate the EMT, proliferation, migration, invasiveness, drug resistance, and metastasis of neighboring cancer cells. Among the regulatory molecules in the interaction between CAFs and cancer cells, lncRNAs are relatively novel biomolecules and are receiving more and more attention from cancer researchers. However, because they have lower expression levels, lower stability, less accurate annotation, and more complicated functional mechanisms compared with protein-coding mRNAs and miRNAs, more difficult and higher barriers lie ahead of lncRNA research [90]. Recently, the development of novel methodologies, such as RNA IP followed by sequencing, high-throughput sequencing cross-linking IP, and chromatin isolation by RNA purification sequencing, has enabled broader and more detailed studies to elucidate the functional molecular mechanisms of lncRNAs [91].

In the clinical aspect, lncRNAs in the tumor microenvironment can be used as potential markers for diagnosis and prognosis and therapeutic targets [92]. LncRNAs, such as *FLJ22447*, *HIPK1-AS*, and *TIRY*, are highly expressed in tumor stroma and are associated with tumor stages or metastatic status. Working within cancer cells, the lncRNAs described above also promote cancer progression and metastasis. *ANRIL* and *DNM3OS* are responsible for resistance to cancer therapy and could be used as predictive markers for therapeutic response. Exosomal lncRNAs, such as *H19*, *CCAL*, and *UCA1*, are also involved in drug resistance, suggesting that these are potential candidates for liquid biopsy. Given that lncRNAs that mediate the interaction between CAFs and cancer cells can promote cancer progression and metastasis, therapeutic targeting of these lncRNAs by RNA interference-mediated lncRNA silencing or inhibition of lncRNA-protein/mRNA/miRNA interactions will be applicable in the clinical treatment of cancer [93].

## Figures and Tables

**Table 1:** LncRNAs regulating the interaction between cancer-associated fibroblasts (CAFs) and cancer cells. EMT: epithelial-to-mesenchymal transition.

LncRNA	Function	Working Target	Reference
*LncRNAs within CAFs*			
*FLJ22447*	Promotes CAF activation and the progression of oral squamous cell carcinoma	IL-33	[64]
*HIPK1-AS*	Promotes CAF activation, inflammation, and the progression of cervical cancer	-	[65]
*TIRY*	Promotes EMT in CAFs and the progression and metastasis of oral squamous cell carcinoma	miR-14/*WNT3*	[66]
*LncRNAs within Cancer Cells*			
*ZEB2NAT*	Promotes the EMT, migration, and invasion of bladder cancer cells	*ZEB2*	[41]
*LINC00092*	Promotes the EMT, glycolytic phenotype, and metastasis of ovarian cancer cells	*PFKFB2*	[70]
*UCA1*	Promotes the glycolysis and invasion of glioma cells	miR-182/*PFKFB2*	[71]
*UCA1*	Promotes the proliferation, migration, invasion, and EMT of colorectal cancer cells	miR-143/*KRAS*	[72]
*CASC9*	Promotes the progression of cervical cancer	miR-215/*TWIST2*	[74]
*HOTAIR*	Promotes the EMT, migration, invasion, and metastasis of breast cancer cells	*EGR1*, *CDK5RAP1*	[75]
*ANRIL*	Promotes the proliferation, cisplatin resistance, and progression of oral squamous cell carcinoma	ABC transporters	[78]
*DNM3OS*	Promotes radiotherapy resistance in esophageal squamous cell carcinoma	-	[79]
*LncRNAs within Exosomes*			
*H19*	Promotes cancer stem cell phenotypes and oxaliplatin resistance in colorectal cancer cells	miR-141/β-catenin	[82]
*CCAL*	Promotes oxaliplatin resistance in colorectal cancer cells	HuR/β-catenin	[84]
*UCA1*	Promotes cisplatin resistance in vulvar squamous cell carcinoma cells	miR-103a/*WEE1*	[85]
*SNHG3*	Promotes proliferation and glycolytic metabolism in breast cancer cells	miR-330/*PKM*	[86]
*Gm26809*	Promotes CAF reprogramming and activation	-	[88]
*lnc-POU3F3*	Promotes CAF reprogramming and activation	IL-6	[89]

## Abbreviations

lncRNAs	Long non-coding RNAs
miRNAs	microRNAs
*KCNQ10T1*	KCNQ1 overlapping transcript 1
PRC2	Polycomb repressive complex 2
*PANDA*	p21-associated ncRNA DNA damage-activated
*TERRA*	Telomeric repeat-containing RNA
*GAS5*	Growth arrest-specific 5
*PTENP1*	PTEN pseudogene 1
ceRNAs	Competing endogenous RNAs
*HOTAIR*	HOX transcript antisense intergenic RNA
H3K4	Lysine 4 of histone 3
*ANRIL*	Antisense non-coding RNA at the INK4 locus
*Fendrr*	Fetal-lethal non-coding developmental regulatory RNA
*AIRN*	Antisense of IGF2R non-protein coding RNA
CAFs	Cancer-associated fibroblasts
qRT-PCR	Quantitative reverse transcription-PCR
GO	Gene ontology
KEGG	Kyoto Encyclopedia of Genes and Genomes
*HIPK1-AS*	HIPK1 antisense RNA
EMT	Epithelial-to-mesenchymal transition
*ZEB2NAT*	ZEB2 natural antisense transcript
*UCA1*	Urothelial carcinoma-associated 1
*CASC9*	Cancer susceptibility candidate 9
IP	Immunoprecipitation
*DNM3OS*	DNM3 opposite strand/antisense RNA
*CCAL*	Colorectal cancer-associated lncRNA
*SNHG3*	Small nucleolar RNA host gene 3
